# Enhancing Infection Prevention Capacity among Health Workers at Faith-Based Health Facilities using a Champion-Led Training Approach

**DOI:** 10.1017/ash.2025.299

**Published:** 2025-09-24

**Authors:** Shillah Nakato, Akankwatsa Dickson, Maureen Kesande, Joseph Ekong

**Affiliations:** 1 Infectious Diseases Institute, Makerere University; 2 Infectious Diseases Institute, Uganda.; 3 Uganda Catholic Medical Bureau

## Abstract

During the COVID-19 pandemic, Uganda’s healthcare system was significantly strained resulting in economic losses and increased morbidity and mortality. implementing rigorous Infection Prevention and Control (IPC) interventions were crucial to safeguard patients and healthcare workers from nosocomial infections and ensure continuity of essential services. Although faith-based health facilities contribute to 15% of Uganda’s healthcare system, most IPC programs have predominantly focused on public health facilities. This study describes a champion-led cascade model for IPC capacity building designed to strengthen IPC at Private Not-for-Profit health facilities bolstering responses to pandemics like COVID-19 and Ebola. Between October 2020 to May 2021, a Champion-led cascade model was implemented in 213 faith-based health facilities in Uganda. We identified health workers from each health facility to participate in a 3-day IPC Training of Trainers (ToT) based on Uganda’s National IPC training package. The training focused on improving knowledge, and practices in establishing IPC leadership at health facilities, developing guidelines, environmental cleaning, hand hygiene, use of personal protective equipment (PPE), screening and isolation, waste management. The trainees underwent a pre and posttest evaluation and were considered to have passed as national trainers if they obtained at least 70% in the post-test evaluation. They were then assigned to champion IPC improvement at one health facility each through monthly mentorship visits. The Mentorship focused on improving IPC practices suchas environmental hygiene, waste management, use of PPE, screening and isolation. Monthly facility IPC assessments were conducted using a digitalized Ministry of health assessment tool. Facilities with low scores were consecutively profiled for targeted quality improvement. We analyzed improvement in IPC knowledge of the champions, frequency of mentored health workers and improvements in IPC capacity at end line versus baseline using Stata 14.0. A total of 240 champions were trained and they cascaded IPC mentorship to 213 faith-based health facilities (Hospitals=17%, primary healthcare facilities=83%). The champions’ average knowledge improved from 36% at pre-test to 70% at post-test, reflecting a 34% improvement. Overall, 2,963 healthcare workers (1,727 females) were trained in 8 months. Average IPC performance at health facilities improved from 38.6%(SD=12.3) at baseline to 51.3% (SD=10.4) (p < 0 .05). IPC improvements were registered in availability of screening and isolation facilities from 6.8% to 8.4% (SD=3.1) at end line, and PPE use from 3.5% to 4% (SD=1.5). Availability of water remained low (1.6% at baseline versus 1.66% at end line).

The champion-led cascade approach facilitated expansion of IPC mentorship to health workers and enhanced IPC capacities in faith-based facilities across the country.

